# Dietary Restriction for The Treatment of Meniere’s Disease

**Published:** 2020-05-31

**Authors:** P De Luca, C Cassandro, M Ralli, FM Gioacchini, R Turchetta, MP Orlando, I Iaccarino, M Cavaliere, E Cassandro, A Scarpa

**Affiliations:** 1 Department of Medicine and Surgery, University of Salerno, Salerno, Italy; 2 Surgical Sciences Department, University of Turin, Turin, Italy; 3 Department of Sense Organs, Sapienza University Rome, Rome, Italy; Center for Hearing and Deafness, University at Buffalo, Buffalo, NY 14214, USA; 4 Ear, Nose, and Throat Unit, Department of Clinical and Molecular Sciences, Polytechnic University of Marche, Ancona, Italy; 5 Department of Sense Organs, University Sapienza, Rome, Italy; 6 AOU San Giovanni e Ruggi D’Aragona, University of Salerno, Italy

**Keywords:** Meniere disease, vertigo, hearing loss, tinnitus

## Abstract

Meniere’s disease (MD) is an idiopathic inner ear disorder characterized by spontaneous recurrent vertigo, fluctuating sensorineural hearing loss (SNHL), aural fullness and tinnitus. Endolymphatic hydrops (EH) of the inner ear is currently considered the pathophysiological mechanisms that underlies typical symptoms of MD. MD diagnosis is based on the criteria of the Baràny Society. There are many therapeutic options for MD, but none is considered effective by the scientific community. The first-line treatment commonly includes dietary modification, as low salt diet and reduction of alcohol and caffeine daily intake.

Although some studies showed a positive effect of these dietary restrictions, even in the prevention of recurrences, currently there is no uniform consensus on their usefulness.

New dietary approach, such SPC-flakes, are being evaluated: further assessments will be needed to validate their use in clinical practice.

## I. INTRODUCTION

Meniere’s disease (MD) is an idiopathic inner ear disorder, first described by Prosper Meniere in 1861, characterized by spontaneous recurrent vertigo, fluctuating sensorineural hearing loss (SNHL), aural fullness and tinnitus [1,2,3]. The main clinical aspect in MD is the recurrence of sudden and unexpected vertigo attacks that are often debilitating and may severely affect quality of life [4,5].

**Fig. 1:**
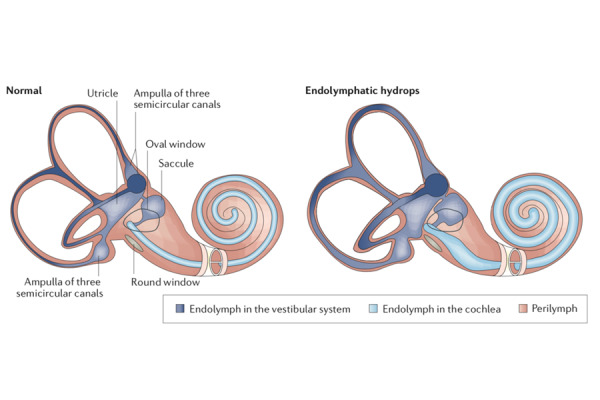
Examples of two labyrinths, one normal and the other with endolymphatic hydrops (EH) EH is a pathological condition in which there is a distention of endolymphatic space by enlargement of endolymphatic volume. From Nakashima et al [3].

Endolymphatic hydrops (EH) of the inner ear is currently considered the pathophysiological mechanisms that underlies typical symptoms of MD. EH seems to be due to an overproduction of endolymph and/or a decrease in the absorption of endolymph as showed by histopathological research [Fig. 1].

MD diagnosis is based on the criteria of the Baràny Society [6].

There are many therapeutic options for MD, but none is considered effective by the scientific community [7] [Table 1].

The first-line treatment commonly includes dietary modification such as restriction of salt, caffeine, alcohol intake and several drugs [8,9].

Concerning the current opinions about which should represent the better dietary management in patients affected by MD, we want to underline the lack of mention about glucose daily intake.

In a recent review from our group, we remarked the possible role played by hyperinsulinemia in subjects affected by MD [10].

We know that the saccule, the main labyrinthine structure affected by pathological damage due to the endolymphatic hydrops, has a large numbers of insulin receptors. This observation was confirmed by the examination of cadaveric subjects and in vivo analysis by cervical Vestibular Evoked Myogenic Potentials (cVEMPs) [11]. Based on this evidence, we suggest to pay special attention in the evaluation of MD patients who present hyperinsulinemia anticipating a MD crisis. So, we think that it is important to define the correct daily intake for patient with MD to reduce recurrences.

In order to prevent crisis, we observed that patients with unilateral MD treated with specially processed cereals that increase endogenous antisecretory factor synthesis compared to patients treated with intravenous glycerol and dexamethasone, showed a reduced number of vertigo attacks and a positive effect on the discomfort generate by tinnitus and quality of life [12].

Several drugs have been proposed for the treatment of MD both for acute attacks (dimenhydrinate, benzodiazepines), and as a prophylactic therapy (betahistine, βblockers, diuretics), although evidence of their efficacy is lacking [3].

When first-line treatment does not offer a satisfactory symptom control, especially for vertigo, more invasive treatments classified into conservative and ablative are recommended [7]. Intratympanic (IT) administration of corticosteroids or gentamicin is commonly used [13,14]. Corticosteroids have been shown to have a lower risk of hearing damage [15,16] but less efficacy in vertigo attack control compared to gentamicin [17,18].

Gentamicin, administered in different doses and timing, has been proven as an effective treatment for vertigo in MD with a potential risk of hearing loss [19,20,21,22].

Controversy remains about the gentamicin dosage and method used. Some physicians favor the use of low-dose gentamicin in which the drug is injected once and further treatments are only performed in cases of recurrent vertigo attacks; other authors prefer high-dose gentamicin, titration or continuous administration in which the drug is injected until vestibular weakness is reached [23,24,25,26,27,28,29,30,31].

However, hearing loss and healthy-side vestibular hypofunction after IT gentamicin administration still represent potential risks of this treatment due to its ablative nature and ototoxicity [32].

Other studies demonstrated that low-dose intratympanic gentamicin can produce a satisfactory control of vertigo attacks after treatment with limited risk of hearing loss [33].

There are some patients with frequent vertigo attacks, progressive hearing loss and persistent annoying tinnitus even through the continuous standard medical treatments; this is called “intractable Meniere’s disease [34].

The consensus is that management of Meniere’s disease should follow a therapeutic escalation from non-invasive medical treatment to surgical treatment that may involve ablation.

Surgery is not to be considered without audiometric and imaging assessment: before considering ablation, ipsi- and controlateral vestibular function should be assessed [35].

Invasive procedures such as vestibular nerve section or labyrinthectomy may be suggested in case of treatment failure.

Recently, an electronic questionnaire formulated by a work group leaded Quaranta and sent to Italian otolaryngologists, showed that in Italy, refractory case of MD are treated initially with IT steroids and, therefore, with gentamicin; in case of failure of IT treatment, vestibular nerve section is the treatment of choice [36,37,38,39,40,41].

In this review article, we would like to consider the evidences for dietary restriction in the treatment of Meniere’s disease.

## II. DIETARY RESTRICTION FOR THE TREATMENT OF MENIERE’S DISEASE

There is no consensus about the first line medical treatment of Meniere’s disease to produce symptomatic improvement and slow the disease progress. Dietary restrictions is among the first line treatment that has been proposed for long. They include low salt diet, abundant water intake, moderate alcohol and caffeine consumption, gluten-free diet, intake of specially processed cereals (SPC-flakes).

### A. Low salt diet

Low salt diet is widely used as a first line treatment option. Daily sodium intake is recommended to be under 2000 mg. The low salt intake is believed to be helpful in lowering endolymphatic pressure; however, this is already to prove [42].

Luxford E. et al. found an improvement in the number and severity of vertigo with dietary modification containing low sodium diet [43]. Beneficial result of dietary sodium restriction has also been documented by Sheahan SL. et al. [44].

Dietary sodium restriction has been advised by many clinicians as a first line treatment for Meniere disease [45]. Also Miyashita et al [46] suggested that low-salt diet should be an effective first line of treatment for patients withe MD; in authors’s opinion, this treatment will have a greater effect, when sodium intake is reduced to less than 3 g/day, due to an increase in the plasma aldosterone concentration that can activate ion transport and absorbing endolymph in the endolymphatic sac.

In a study by Acharya et al. there is no evidence of benefit by dietary salt restriction in terms of hearing improvement, number of vertigo, severity of vertigo and tinnitus score [47].

De Ru and Heerens suggested that it is not sodium that should be avoided but potassium [48].

However, strong evidence that salt restriction is beneficial does not exist.

### B. Abundant water intake

Naganuma et al [49] proposed time-series study with historical control regarding the water intake therapy for patients with MD that demonstrates that water intake therapy could improve and prevent hearing loss compared to other conventional therapies.

Hearing ability and relieved vertigo are improved in these patients by the increased drinking water and decrease of plasma ADH level.

A group leaded by Kitahara [50] confirmed that abundant water intake (35 mL/Kg/day as specified by Naganuma) can be a feasible treatment in Meniere’s disease.

### C. Alcohol and caffeine consumption

Regarding alcohol, many clinicians recommend to avoid or reduce its consumption. A recent work by Sanchez-Sellero et al [51] evaluated the possibility that alcohol consumption delays the age at onset of Menière’s disease; the authors explained this by the inhibitory effect of alcohol on hypothalamic production of vasopressin: a lower release of this neurormone increases diuresis while decreasing endolymphatic pressure.

However, actually the literature doesn’t offer review to evaluate if alcohol consumption is more prevalent or more intense in patients with MD.

Another substance highly considered in the preventive treatment of MD is caffeine, an alkaloid present in many foods and as an additive in “cola-type” drinks and “energy drinks”.

Pharmacological effect of caffein might include antagonistic effects on adenosine receptors, renal effects as diuresis and natriuresis, activation of renin-angiotensin-aldosterone system, release of corticosteroids by adrenal cortes and catecholamines drama adrenal medulla [52].

Also Sanchez-Sellero [53] observed that caffeine should be considered as a precipitating factor for the onset of symptoms in people predisposed to developing Menière’s disease. The authors suggested that it should be recommended to reduce caffeine intake in those population groups with higher risk of Menière’s disease.

### D. Gluten-free diet

Recently other possible dietary modifications have been linked to Meniere’s disease’s treatment.

Di Berardino et al [54] reported a case of remitted unilateral Meniere disease after 6 months of a restrictive gluten-free diet. The same authors were the first to report a gliadin skin test in 33 patients with definite MD [55] and the first to observed an altered intestinal permeability in symptomatic MD patients.

Further studies will be necessary to assess if the investigation of intestinal permeability and fecal calprotectin can be added to the current clinical practice, aimed at identifying those MD patients who would benefit from deprivation diets.

### E. Specially processed cereals (SPC)

Recently, the intake of antisecretory factor (AF)-inducing specially processed cereals (SPC)-flakes has been proposed as complementary therapy for Meniere’s disease.

Antisecretory factor (AF) is a 41 kDa protein originally isolated due to its ability to inhibit experimental diarrhoea; the specific effect of endogenous AF is not completely understood, but it seems to modulate water and ion transport.

The intake of specially processed cereals (SPC-flakes) results in an increase of AF activity in plasma [56]. Hanner et al hypothesized that an increased AF activity could positively influence the course of MD: their work demonstrated that the intake of SPC-flakes not only significantly reduced vertigo in a half of the patients, but was also related to an increase level of active AF in plasma and positive clinical outcome.

Treatment with SPC appears to be well tolerated by most patients [57] without any complications and, in most of studies, more than half of the study cohort reported subjective improvement in functional level.

On the contrary, Ingvardsen and Klokker [58] observed that antisecretory factor-inducing (AF) SPC were not shown to significantly improve the functional level in patients with MD.

## III. CONCLUSION

Many clinicians recommend to reduce the intake of salt, caffeine and alcohol for the therapy of Meniere’s disease.

Although some studies showed a positive effect of dietary restrictions, even in the prevention of recurrences, currently there is no uniform consensus on their usefulness.

New dietary approach, such SPC-flakes, are being evaluated: further assessments will be needed to validate their use in clinical practice.

## Figures and Tables

**Table 1:** First-line and second-line therapy for Meniere’s disease.

First-line Treatment	Dietary modification	Drugs administration
	
	Low salt diet	Diuretics
	
	Abundant water intake	Steroids
	
	Alcohol and caffeine consumption	Bethaistine
	
	Gluten-free diet	Dimenhydrinate
	
	Specially processed cereals	Benzodiazepines
	
**Second-line Treatment**	**Conservative procedures**	**Ablative procedures**
	
	Intratympanic steroids	Intratympanic gentamicin
	
	Endolymphatic sac surgery	Vestibular neurectomy
	
	Pressure pulse treatment	Labyrinthectomy
